# The Principles and Practice of Obstetricy

**Published:** 1835-01-01

**Authors:** 


The Principles and Practice of Obstetricy. By *James Blundell*, M.D. Professor of Obstetricy at Guy's Hospital. To which are added Notes and Illustrations, by *Thomas Castle*, M.D. F.L.S. London, 1834. 8vo. pp. 838.

The lectures of the learned Professor, as they appeared in the Lancet, in the years 1830-31, form the foundation of this large and valuable treatise, the subjects of it being merely re-arranged, subdivided, and commented on by the editor. It is well known to the whole medical profession of this country that the author of this work not only succeeded to the chair of one of the most eminent lecturers on midwifery this country has ever produced, but that he has been one of the chief supporters of the celebrity of the medical school at Guy's Hospital for the last twenty years. From this conspicuous and useful station he has just retired, and it is probable, from the state of his health and circumstances that the great mass of information contained in this volume would have been lost, or at least would have been published in a much more imperfect form had this volume never appeared. It is impossible to rise from a perusal of these lectures without being impressed with respect for Dr. Blundell. They afford ample proofs that he is a keen and cautious observer, a sincere lover of truth, remarkably free from prejudices and prepossessions of every description, and liberal and just to all. While we state this we cannot conscientiously give our approbation to every part of the work, nor do we think upon the whole that it will contribute much to the dignity or the improvement of obstetrical medicine. There is often a straining at originality where there is no new idea to express; the language is often ill-suited to the subject, being flowery and poetical when it should have been peculiarly clear and concise: in some parts there are long passages, bordering almost upon indelicacy; which is a great defect in writers and lecturers upon midwifery and the diseases of females. In proof of the latter fact see p. 70, 1, 2, and 3.

We had marked a long passage for quotation, but we really were unwilling to sully our pages with language which we are sure Dr. Blundell himself cannot approve of, when he reflects, but for a moment, on its grossness—we had almost used the term OBSCENITY.

The work of Dr. Blundell is divided into five parts. 1. The Anatomy of the Female System. 2. Physiology of the Female System. 3. Signs and Diseases of Pregnancy. 4. The Act of Delivery. 5. After-management of the Puerperal State. In the first part there is no room for originality. The account of the structure of the uterine organs is clear and correct, and it is illustrated with several wood-cuts well executed.

Menstruation, conception, sterility, and embryology, are the most important sections in the second part of the work.

Respecting the probable use of menstruation, Dr. Blundell observes:—

"Much has been written, and many points assiduously discussed, concerning the use of menstruation; but it appears most probable to me, as the discharge only flows during the child-bearing period of life, that it is associated in the way of cause and effect, with aptitude for impregnation: before puberty there is no menstruation, and after a term of some thirty years, when the powers of fecundity are lost, the menses are found to cease more or less suddenly; impregnation, however, may certainly occur, though the catamenia have never appeared." 53.

Dr. Blundell has said nothing about the influence of the ovaria on the uterine system at the period of menstruation, although we think it is demonstrated by the observations of Dr. Lee, that all the phenomena depend on certain changes which take place in the Graafian vesicles at each monthly period.—*See Cyclopædia of Practical Medicine, article Ovaria.*

Menstruation, it is said by some, keeps and preserves the uterus in a state fit for impregnation. If so, how do women fall with child who have never menstruated? And it is a subject of daily observation, that women who are suckling become pregnant though they have not menstruated since the former conception.

Our author is of opinion that menstruation sometimes takes place during pregnancy.

"Although during the child-bearing period of life, women menstruate, this action is entirely arrested during pregnancy and suckling, there being, however, exceptions to the general rule. Some women menstruate during the first months of gestation, nay, perhaps, in some rare instances throughout the whole process; in most cases, however, it ceases, and also ceases during suckling, though in the latter process, it is not unfrequently renewed at the end of ten or twelve months, although the suckling be continued still; and hence we must not hastily conclude that a woman is not pregnant, merely because she menstruates, for although doubts, may be raised respecting the continuance of the catamenia during the whole term of gestation, yet I have repeatedly met with cases of pregnancy, in which the catamenia have continued to flow during the first two or three months;* indeed, this, notwithstanding Denman's assertion to the contrary, may, I think, be looked upon as by no means very uncommon." 53.

As the deciduous membrane coats the inner surface of the uterus, and the

* This militates against the idea that menstruation depends upon certain conditions of the Graafian vesicles.

os uteri is closed with a plug of thick jelly, it is difficult to understand how this can take place. If it is real menstruation, it must be from the upper part of the vagina or cervix uteri. Further investigation upon this subject is required.

The second section contains a history of the phenomena of conception and impregnation.

The researches of Dr. Haighton on this subject are known to all. Dr. Blundell has also made laborious investigations into this department of physiology, and has determined several points which were before doubtful.

His experiments on rabbits, to prove that, in the animal at least, the contact of the male semen with female ova was necessary for fecundation, are well known, and are to be found in the second volume of this series, pp. 408. We shall give the inferences which Dr. B. has drawn from his experiments.

> "First:—From these experiments we may infer, that in the rabbit, corpora lutea may form independently of the full excitement of the generative actions, and, therefore, that in this animal they are not the certain evidences of impregnation. By the corpora lutea, I understand those appearances, which, when impregnation is effected, seem to shew themselves invariably in that part of the ovary from which the rudiments have escaped.
>
> Secondly:—We may also infer, that mere absorption of the semen from the vagina by means of the lymphatics, is insufficient for the purposes of formation. In one of the vaginal experiments, the access of the semen to the rudiments being intercepted, impregnation could not be accomplished, though the animal admitted the male as many as fifty times, mostly at intervals of two or three days, or more. This doe, a remarkably fine one of her age, was a great favorite with her polygamous husband; but it appeared, after death, that notwithstanding all these attempts, no fœtuses could form—the corpora lutea were generated—the wombs were evolved—the water, as usual, collected in the uterine cavities, but this was all—the access of the semen to the rudiments was intercepted at the top of the vagina, and impregnation could not be effected. Yet it is evident that much of the male fluid must have been deposited in the vagina, and absorbed by the veins or the lymphatics." 64.

Dr. B. is of opinion that the fecundating rudiments meet not in the ovaria, nor in the fallopian tubes, but in the uterus.

But we find it impossible to glance at a fiftieth part of the volume before us, without occupying the space of two or three long articles in our Journal. Considering, too, that the substance of these lectures has long ago circulated widely among the profession, through the medium of our contemporary the Lancet, we deem it unnecessary to attempt anything like an analysis, while criticism would be just setting the opinion of an anonymous writer against that of an acknowledged name. We shall merely remark that the text and the notes form a mine of gold—a treasure of literature, science, and practical knowledge, for the student, which it would be suicidal madness in him to neglect, or fail to have constantly in his possession for reference.

There is a section, however, towards the close of the work, which we are unwilling to pass unnoticed:—it is termed—

### Hidrosis, or Hidrotic Fever.

This, though not a frequent disease, is considered by Dr. B. as one of considerable importance. The severer varieties are dangerous—and the malignant types almost invariably fatal. It sometimes commences even before parturition has taken place, but more frequently during the first eight or nine days after delivery.

"The disease opens usually, if not always, with a shudder more or less severe, and so far resembles puerperal and other fevers; sometimes the shudder is slight, lasting for three or four minutes only, and attracting but very little attention, while in other cases the patient may shake, as if she was in an ague fit. In general, this shuddering is accompanied with a sensation of cold, which is occasionally intense; while in other cases, the feeling of coldness is slight, or perhaps wanting altogether; and I have been told by the attendants, that the patient has exclaimed—'I am so cold,' and called for more covering, though the flesh has felt warm to the hand of the nurse.

The shuddering and the feeling of coldness are not always in proportion to each other: thus, the patient may shake violently, the sensation of cold being slight, or, she may complain much of the cold, without suffering a smart attack of the shudders: as in cases of puerperal fever, so also in this disease, there is sometimes only one attack; but we may observe occasionally, three or four occurring at uncertain intervals of hours or days—nay, in the same patient, where the disease continues in its lingering form for a period of several weeks, there may be a great many rigors, and this may now and then tend to observe the quotidian period, though the patient may suffer two or three attacks in the course of a day, at irregular intervals. In this disease, further, there is more or less disposition to sweats and heats, combined, which constitutes a very characteristic symptom. These sweats are, I think, at first more fluid, but they afterwards become more clammy, especially towards the close of the disease, during the last few hours. In some cases we find them to be sparing, while in others, especially in the more malignant varieties, they are surprisingly profuse. But whether they are sparing or copious, fluid or viscid, they are never critical—that is, they do not remove or effectually relieve the disease, to the great disappointment of the practitioner, and they may, I think, be not inaccurately described as *sweats of distress* in the system." 772.

The rapidity of changes in the pulse is remarkable. From 90 it will suddenly rise to 140 or 150, without any evident cause. Upon the whole, it is very unlike the pulse of puerperal fever, in any thing but its frequency.

"In hidrotic fever, there is not unfrequently a morbid state of the nervous system, which shows itself in a certain quickness of manner, a rapidity of utterance, or a disposition wayward, pettish, or passionate. Sometimes, also, the patient becomes the subject of whimsical impulses, either of a comic or tragic character, so that there is an evident tendency to puerperal mania, which may ultimately, though not generally, occur. On the other hand, the patient's manner is now and then marked with a sort of forced calmness, and in some cases there is no very obvious disorder of the nervous system, for these symptoms are not constant." 773.

The lacteal secretion is often disturbed; being suspended or changed. There is sometimes very little pain in this fever; but, in one stage or other, there will usually be felt uneasiness about the pelvis;—occasionally in other parts. Tympanitis and sub-tympanitis not unfrequently occur in this fever, especially towards the close of the disease. The blood is sometimes greatly inflamed—sometimes very obscurely so. It terminates by resolution, collapse, or conversion into some other affection.

"Sometimes, in the milder form especially, the disease is brought to its close by a gradual retreat of the symptoms, so that day after day it gets milder, till the patient ultimately gets well. But in the severer forms of hidrosis, even those varieties of it which do not appear very formidable at the outset, there is, I suspect, always a pertinaceous tendency to collapse, the strength sometimes giving way very rapidly; say, in the course of a few hours; as if the patient had been poisoned. While in other cases, though the system holds out for three, four, five, or six days; yet the powers are at length laid prostrate, and the patient at last sinks. The complete collapse is marked by the usual symptoms —a pulse of perhaps 117 a minute, small, and easily stopped by compression— a corpse-like coldness of the hands and feet—breathing more or less laborious, sometimes very much so; and occasionally a tympanitic affection of the abdomen.

When the disease terminates in the third mode (if termination it can be called,) it is converted into some other affection, and the patient is assailed with puerperal mania, abscess of the breasts, phlegmasia dolens, &c.; the form and seat of the affection changing, although the nature of it probably remains the same." 774.

Dr. B. divides the hidrotic fever into no less than seven varieties—the ultra-malignant—the malignant—the acute—the lingering—the mutable—the fugaceous—the remittent. We confess that these subdivisions appear to us rather superfine. The splitting of diseases according to their *degrees of severity*, has always seemed to us a very difficult mode of distinction. The mere plus vel minus of intensity in a disorder is any thing but an accurate, or at all events, practicable diagnosis. As no two cases of typhus fever, for example, will be found precisely alike, in severity or even in duration, and as more than fifty millions of men have had typhus, we might thus be justified in dividing fever into fifty million of varieties. Our author, however, dedicates a section or head to each of the seven varieties of hidrosis, for which we must refer to the book itself. The description of the seventh variety will shew that the distinctions in question are not very stable in their foundations.

"There is, too, a seventh variety of the hidrosis, the intermittent or remittent. *I suspect, that this variety of the disease is in nature different from the genuine hidrosis, but am not yet determined on the point;* sometimes there is a single paroxysm only, consisting of chill, heat, and sweat; probably identical with the weed, or ephemera so well described by Burns. In other cases, we have repeated paroxysms occurring at irregular intervals, two or three times in the course of the day, and this for days together, or there may be repeated attacks which observe the quotidian period, and regularly commence with a chill. Perhaps the two last variations here mentioned, the quotidian I mean, and the irregular, may be referred to a form of the disease already considered; namely, the lingering hidrosis." 781.

In all these varieties, our author *suspects* that the "patients are liable to attacks of retching and vomiting, with bilious diarrhœa—while the lochia are pale, offensive, and sometimes suspended." We hardly suspected such loose statements from a man like Dr. Blundell, after the minute manner in which he handled the subject of diagnosis.

In respect to etiology, our author seems to think that parturition is the great predisposing cause—that it is connected with failure of the mammary secretion, though not, perhaps, invariably so—that flooding, whether before or after delivery, has a tendency to induce the disease—that the induction of premature delivery has a similar tendency, together with the rude detachment of the secundines. He suspects, too, that lodgment of putrescent pieces of the placenta has given rise to hidrosis.

" As to the more immediate cause of hidrotic fever, my mind is in doubt. Some varieties of it at least, especially the lingering and the mutable, appear to arise from the inflammation of the veins of the uterus, so well investigated by Dr. Robert Lee. Upon this hypothesis several of the phenomena may, I think, be explained ; and that correspondence is the more remarkable, as this supposition has in no way whatever influenced the characters which I have given of the disease, as they had been all marked out, more or less distinctly in my adversaria, before I was acquainted with his valuable labours in this part of morbid anatomy." 783.

It may be distinguished, he observes, from typhus by its connexion with delivery—by the characteristic sweats which do not prove critical—by its running its course sometimes so rapidly, say, in three or four days—by the absence of black crusts or aphthous redness so frequent in typhus—and by the general aspect, which is altogether different.

" From puerperal fever the hidrotic fever may be distinguished in the variableness of the pulse, which is remarkably observable in some hidrotic cases, as well as in the bounding, and softness, and roundness which is sometimes observed in the obscurity of the abdominal symptoms, which are certainly not prominent nor perhaps essential in the characteristic sweats, and perhaps, I may add, the general warmth of the body ; in the failure or total suppression of the milk ; in the milder cases, in the lingering duration of this disease, which even in the acuter form may last five or six days, and then prove fatal ; also under some varieties of hidrosis in the first attack of the disease, not commencing till the fifth or sixth day of delivery, in the formation of a thick white crust on the tongue, (and under the mutable variety) in the disposition to the attack of other diseases already enumerated." 784.

The prognosis is, upon the whole, unfavourable, and but little is said as to the treatment. In the malignant and ultra-malignant varieties, he fears that little can be done by medicine. In the latter variety the patient gives way as rapidly as in cases of virulent poison or malignant cholera. Stimulants are indicated—and in the malignant form he queries venesection and calomel. In the third variety, (acute hidrosis,) he would be inclined " to throw in mercury immediately," keeping the head cool, the feet warm, the bowels open, and the liver in action.

The few specks which we have noticed on the face of this fair volume were scarcely deserving of censure or criticism. The defects and the errors are probably fewer than in any other work of equal extent and importance in the English language. This is no mean praise, but it is well earned. Dr. Castle, the editor, has done his duty with care and judgment. The notes are often copious and judicious, referring to many recent authorities which the lecturer, in the ripeness of experience and in the turmoil of avocation, had probably neither leisure nor inclination to consult. We need scarcely add that the work, as it now stands, will be a class-book, not only for obstetric students, but practitioners of every grade and distinction.

